# Elimination of Spirochaetes in the Urine, in Spirochetosis Icterohemorrhagica

**Published:** 1919

**Authors:** 


**_Elimination of Spirochetes in the Urine, in Spirochetosis Icterohemorrhagica_**. By MM. GARNIER and J. REILLY. *La Presse Médicale*, October 3, 1916.

The detection of characteristic spirochetes in the urine of the patients is the most conclusive evidence for the diagnosis of infective jaundice.

The presence of the spirochetes in the blood circulation can only be detected by injection to guinea-pigs, and the virulence of blood disappears rapidly after the first days of the disease.

The research of immunisins in the blood serum can only be made after the fourteenth day of the illness; these substances fail in slight attacks of infective jaundice.

Agglutination has given good results, but it needs a culture and the use of the ultra-microscope.

On the contrary, the research of the spirochetes in the urine is a very simple and safe method.

(1) Research of the Spirochetes in the Urine.

The meatus being washed carefully, urine is collected in a sterilized beaker; 10 or 12 cc. of urine are centrifugalized, and a drop of the deposit spread out on a glass slide.

Two methods may be resorted to: either the staining of the micro-organisms themselves, or the dyeing of the background.

In this last case, both India ink or Congo Rouge may be used. A drop of dye is deposited on the drop of centrifugate, both are spread out rapidly and dried carefully by moderate heating. The addition of a few drops of hydrochlorid alcohol at 1 per cent. dyes all the fluid background in blue, while the spirochetes and epithelial cells existing in the urine remain unstained.

For dyeing the spirochetes themselves, different well-known dyes may be used, but the best results are obtained with the silver impregnation method; they give the spirochetes a dark brownish color, thus rendering them easy to detect.

(2) The spirochetes appear in the urine around the tenth day of the illness. Between the fifteenth and twenty-third day, frequent examinations of the urine must take place. A single negative examination is not conclusive for a negative diagnosis.

Generally the spirochetes appear in the urine at the beginning of the relapse of fever, but both phenomena do not seem to be directly connected.

(3) Characters of the Elimination.

The eliminated spirochetes increase rapidly in number for a few days. For one or two days the elimination is at its climax, and the micro-organisms are so numerous as to form real heaps in the preparations. The elimination lasts from 5 to 10 days on an average, but it is not constant during all that tine. Exceptionally it has been noted by the authors to last only one day in one case, and as long as 20 days and more in others.

(4) Morphological Characters of the Spirochetes.

At the onset of the elimination, the spirochetes are only slightly undulous, hooked in the form of a C or an S. Later on degenerated forms are met with.

(5) Two varieties of spirochetes, different from spirocheta icterohemorrhagiae, may be met with in the urine of patients and healthy subjects.

One of these organisms is most usually present in the preputial smegma; it presents very fine and numerous undulations and resembles the degenerated forms of spirocheta icterohemorrhagiae.

The other variety has all the morphological characters of spirocheta icterohemorrhagiae; it is met with frequently when the glans is inflamed.

The presence of these spirochetes may be avoided by taking special care in collecting the urine before examination and washing thoroughly the meatus.

(6) The urine of jaundiced patients is sometimes, but very rarely in fact, virulent during the first fortnight of the disease. During that period, injections of large quantities of urine, as large as 250 cc., do not affect the guinea-pigs.

Normally the virulence of the urine lasts from the 15th to the 23rd day. During that time, injections of urine determine fatal attacks of infective jaundice in guinea-pigs, provided the injected urine contains spirochetes.

Later on, if the urine contains degenerated forms of spirochetes, these still prove capable of determining attacks of infective jaundice in the injected guinea-pigs, but only slight attacks, generally followed by recovery. Thus the virulence of urine appears intimately connected with the elimination of the spirochetes. The organisms eliminated at first possess a more marked virulence than those passing at the climax of elimination.

(7) Postmortem histological examinations of the kidneys in fatal cases show the way in which the micro-organisms are eliminated. At the beginning of the disease the spirochetes are found in the preparations all gathered around the convoluted tubes. No organisms at all exist at first inside those tubes. This explains why they are not met with in the urine until the 14th day of the disease.

Later on, during the period extending from the 14th to the 23rd day of the illness, the spirochetes are seen to have passed through the walls of the convoluted tubes, they are met with inside the cells, but especially in the lumen of the tubes.

Never are the glomeruli infected with the micro-organisms.

This may be compared to some observations made in cases of secondary syphilis. The treponema never exist in the glomeruli, whereas they are found very numerous in the epithelial cells of the convoluted tubes, and especially inside those tubes. The elimination of the treponema is identical to that of the spirochetes. Syphilis and infective jaundice must therefore be considered as comparable.

On the contrary, in streptococcic and staphylococcic infection the micro-organisms collect in the glomeruli.

(8) The following explanation may be given of the fact that the spirochetes are not found in the urine until the 14th day. By this time the biliary salts have lessened in the urine, and their very marked " lytic " action upon the spirochetes diminishes also, thus rendering possible the passage of these micro-organisms.

Later on the immunisins developing in the blood serum bring on the recovery from the disease, and gradual subsiding in the virulence of the spirochetes.

